# A Rare Case of Streptococcus dysgalactiae Subsp. Dysgalactiae Human Zoonotic Infection

**DOI:** 10.7759/cureus.2901

**Published:** 2018-07-01

**Authors:** Suma Sri Chennapragada, Kamleshun Ramphul, Ben J Barnett, Stephanie G Mejias, Petras Lohana

**Affiliations:** 1 Medical Student, Osmania Medical College, Hyderabad , IND; 2 Pediatrics, Shanghai Jiao Tong University School of Medicine/Shanghai Xin Hua Hospital, Shanghai, CHN; 3 Internal Medicine, The University of Texas Health Science Center - Medical School, Houston, USA; 4 Pediatrics, The University Iberoamericana Unibe School of Medicine/Robert Reid Cabral Children's Hospital, Santo Domingo, DOM; 5 Medicine, Liaquat University of Medical and Health Sciences Hospital, Karachi, PAK

**Keywords:** streptococcus dysgalactiae subspecies equisimilus, streptococcus dysgalactiae subspecies dysgalactiae

## Abstract

Streptococcus dysgalactiae has two main subspecies: Streptococcus dysgalactiae subsp. equisimilus (SDSE) and Streptococcus dysgalactiae subsp. dysgalactiae (SDSD). The vast majority of human infections belonging to Streptococcus dysgalactiae have been associated with SDSE. There are only three cases of SDSD found in humans in literature. We present a case of SDSD cellulitis and bacteremia in a 49-year-old female patient from Houston, Texas with no major exposure to animals or trauma.

## Introduction

Since its inception, Streptococcal taxonomy and classification have been subjected to a wide range of modifications. Due to the advances in the precise identification of various streptococcal species and subspecies by means of Verigene and other isolation methods, it has become important to recognize and understand their classifications. These breakthroughs have also led to the identification of rare zoonotic infections in humans by organisms previously known to cause animal infections only. One such organism is Streptococcus dysgalactiae subsp. dysgalactiae.

Streptococcus dysgalactiae was subdivided into two main subspecies in 1996: Streptococcus dysgalactiae subsp. equisimilus (SDSE) and Streptococcus dysgalactiae subsp. dysgalactiae (SDSD). The former expresses Lancefield group C or G antigen while the latter has Lancefield group C or L antigen [1]. The vast majority of human infections belonging to Streptococcus dysgalactiae have been associated with SDSE including pharyngitis, skin and soft tissue infections, bacteremia, and endocarditis. There are only three cases of SDSD found in humans in literature. We present a case of Streptococcus dysgalactiae subsp. dysgalactiae cellulitis and bacteremia in a 49-year-old female patient from Houston, Texas.

## Case presentation

A 49-year-old morbidly obese African-American female patient presented to the hospital with an initial complaint of fever, pain, redness, swelling and discharge in her left lower extremity. The pain was progressive and worsened to 10 out of 10 on the pain scale. It aggravated with movement and on weight bearing. There were no alleviating factors. She denied any recent history of trauma to her leg, chest pain, shortness of breath or any history of prolonged immobilization.

Her past medical history included morbid obesity with a Basal Metabolic Index (BMI) of 85. She also has a history of hypertension, lymphedema, hypersensitivity lung disease, obstructive sleep apnea, and chronic gastroesophageal reflux disease. Her past medical records showed a previous hospitalization for group G and beta-hemolytic streptococcal bacteremia three years ago that was properly treated.

Her family history included hypertension in her father and type two diabetes mellitus in her mother. She denied any consumption of alcoholic and tobacco products or any recreational drug use.

Physical examination showed a morbidly obese patient with a temperature of 101.4 F. Her heart rate was 120 beats per minute and respiratory rate of 33 per minute. Her oxygen saturation at normal air was 91%. Bilateral non-pitting lymphedema in both lower extremities was observed. Her left lower extremity showed erythema and swelling in her calf region with significant serosanguinous discharge. She had a restricted range of motion in her left lower extremity.

The patient was admitted for further investigations. Her complete blood count showed significant leukocytosis of 25,600 cells/mm^3 ^with bandemia, elevated procalcitonin (PCT) levels of 8.33 ng/mL, elevated C-reactive protein (CRP) of 348.0 mg/L suggesting an acute infectious process. Her creatinine level was elevated at 1.22 mg/dL compared to her previous laboratory results of 0.8-0.9 mg/dL. She also showed signs of liver dysfunction with an elevated aspartate aminotransferase (AST) of 56 U/L, alkaline phosphatase (ALP) of 144 U/L, and of coagulopathy with an international normalized ratio (INR) at 1.21.

Left lower extremity Doppler ultrasound showed no evidence of deep venous thrombosis with limited evaluation for popliteal vein compression due to edema. There was Doppler flow within the popliteal vein. Significant edema was identified in the left popliteal fossa without any underlying abscess. Two separate blood cultures were tested positive for Streptococcus dysgalactiae subsp. dysgalactiae. The urine cultures grew no organisms.

The patient was administered a loading dose of Vancomycin in the emergency department and later started on Cefepime. Proper fluid hydration was included in her treatment plans. She was then shifted to Ceftriaxone when her blood cultures came back positive for SDSD. A transesophageal echo was ordered to rule out endocarditis and came back negative for vegetations. Her condition improved slowly as her white blood cell count went from 26,000 cells/mm^3^ to 14,000 cells/mm^3^ and other inflammatory markers such as PCT were back to normal. The pain, erythema, and edema in her left lower extremity also improved gradually.

She was discharged after one week of inpatient treatment at her own request and prescribed another one week of ceftriaxone that she received intravenously at her home. Her follow-up after two weeks showed major improvement.

## Discussion

Streptococci have been traditionally classified as alpha hemolytic, beta-hemolytic and gamma hemolytic. The beta-hemolytic streptococci are further classified according to Lancefield grouping from group A to V, excluding I and J. Streptococcus dysgalactiae forms part of the Lance field group C. SDSE has been associated mostly with humans and SDSD with animals [2-3]. Literature encompassing discussions of the characteristics and pathogenesis of SDSD is very limited.

A study conducted by Alves-Barroco et al. evaluated the in vitro and in vivo potential of SDSD to adhere to human cells of the respiratory tract [4]. They found that the organism could be internalized into those cells by an active transport mechanism and thus proved that SDSD had zoonotic infective capabilities in vertebrate hosts including humans. A thorough search of the literature showed three main cases of SDSD infections. Koh et al. presented a case of SDSD cellulitis after having been in contact with raw fish, while Park et al. reported an SDSD-based prosthetic joint infection after knee arthroplasty [5-6]. In 2015, Jordal et al. found out that their patient had an infective endocarditis from SDSD infection [7]. Both patients presented by Koh et al. and Jordal et al. had some animal exposure. The patient of Park et al. had no direct animal exposure but had been on chronic treatment with a disease-modifying antirheumatic drug (methotrexate ) which was believed to have predisposed him to the condition.

Our patient had no direct injury or trauma from any animal. The only possible close contact she had was with her neighbor’s dog. She denied having any pets. Our patient’s morbid obesity and lymphedema could have predisposed her to infection, similar to one of the previously reported cases. The infection in our patient showed sensitivity to beta-lactam antibiotics and has improved with therapy. The previously reported three cases had also shown sensitivity to beta-lactam antibiotics.

## Conclusions

There are only three published cases of Streptococcus dysgalactiae subsp. dysgalactiae infection in humans. We report the fourth case in a patient with no history of trauma from an animal but with several predisposing factors. She responded well to beta-lactam therapy for the infection.
